# Designing of dual inhibitors for GSK-3β and CDK5: Virtual screening and *in vitro* biological activities study

**DOI:** 10.18632/oncotarget.15085

**Published:** 2017-02-04

**Authors:** Hongbo Xie, Haixia Wen, Denan Zhang, Lei Liu, Bo Liu, Qiuqi Liu, Qing Jin, Kehui Ke, Ming Hu, Xiujie Chen

**Affiliations:** ^1^ Department of Pharmacogenomics, College of Bioinformatics Science and Technology, Harbin Medical University, Harbin 150086, P. R. China; ^2^ Department of Physiology, Harbin Medical University, Harbin 150086, P. R. China

**Keywords:** Alzheimer's disease, GSK-3β, CDK5, multi-target

## Abstract

Alzheimer's disease is a multifactorial neurodegenerative disorder with many drug targets contributing to its etiology. Despite the devastating effects of this disease, therapeutic methods for treating Alzheimer's disease remain limited. The multifactorial nature of Alzheimer's disease strongly supports a multi-target rationale as a drug design strategy. Glycogen synthase kinase-3 beta and cyclin-dependent kinase 5 have been identified as being involved in the pathological hyperphosphorylation of tau proteins, which leads to the formation of neurofibrillary tangles and causes Alzheimer's disease. In this study, using a molecular docking method to screen a virtual library, we discovered molecules that can simultaneously inhibit Glycogen synthase kinase-3 beta and cyclin-dependent kinase 5 as lead compounds for the treatment of Alzheimer's disease. The docking results revealed the key residues in the substrate binding sites of both Glycogen synthase kinase-3 beta and cyclin-dependent kinase 5. A receiver operating characteristic curve indicated that the docking model consistently and selectively scored the majority of active compounds above decoys. The pre-treatment of cells with screened compounds protected them against A*β*_25-35_- induced cell death by up to 80%. Collectively, these findings suggest that some compounds have potential to be promising multifunctional agents for Alzheimer's disease treatment.

## INTRODUCTION

Alzheimer's disease (AD) is the most common neurodegenerative disorder and the most prevalent cause of dementia with ageing. The etiology of this disease is rather complex and not completely understood, but certain indicators such as low levels of acetylcholine, *β*-amyloid (A*β*) deposits, tau protein aggregation, neurofibrillary tangles, oxidative stress, and the dyshomeostasis of biometals are considered the cause of the pathogenesis [1–4]. Designing drugs with a specific multi-target profile is a promising approach to multifactorial illnesses because the simultaneous modulation of multiple targets in a biological network is beneficial in treating a complex disease. With the development of polypharmacology, the strategy of developing multi-target drugs has become an active field, and approximately 20 multi-target drugs have been approved or are in advanced development stages [5]. Therefore, the complex etiology of AD has encouraged active research on multi-target AD drugs.

Glycogen synthase kinase-3 beta (GSK-3*β*) is a proline-directed serine/threonine kinase that is responsible for the phosphorylation of a variety of cellular substrates [6]. GSK-3*β* is involved in the regulation of a wide range of cellular processes, including metabolism, cell proliferation, cardiac hypertrophy, oncogenesis and apoptosis. Although GSK-3*β* is perhaps best known as a potential drug target for metabolic conditions such as type-2 diabetes and insulin resistance due to the effects of this enzyme on glycogen metabolism, GSK-3*β* is highly expressed in the brain and is linked to a variety of central nervous system (CNS) disease states, including AD, Huntington's disease and stroke [7, 8].

There is strong evidence that GSK-3*β* co-localizes preferentially with NFTs. GSK-3*β* is active in pre-tangle neurons and contributes to the formation of paired helical filaments (PHFs) in the AD brain [9]. GSK-3*β* has been shown to phosphorylate tau protein at some of the sites that are hyperphosphorylated in PHFs both in transfected mammalian neuronal cells and *in vivo*. In addition to its role in tau protein phosphorylation, GSK-3*β* is also involved in regulating other AD-related mechanisms.

Cyclin-dependent kinase 5 (CDK5) is an atypical and essential member of the CDK family of proline-directed serine/threonine kinases with no evident role in cell cycle progression. CDK5 is an essential neuro-differentiation and neuro-protective role in normal neuronal physiology, that is directly linked to multiple neurological diseases, such as AD, Parkinson's disease and Huntington's disease [10]. The activation of CDK5 is triggered by the binding of the regulatory subunits p35 or p39 [11]. The CDK5/p35 complex could hyperphosphorylates tau protein and reduces the association of tau protein with microtubules, resulting in cytoskeletal alterations and neuronal apoptosis. This phosphorylation has been described as a key point in controlling the activation of CDK5 [12–14]. It has been observed in cellular experimental models that A*β* stimulates the cleavage of p35 to p25, and the inhibition of CDK5 reduces A*β*-evoked cell death. Moreover, a post-mortem analysis of the brain preparations from AD patients indicates an accumulation of p25 and an increase in CDK5 activity [15]. Furthermore, CDK5 has been shown to potentiate tau protein phosphorylation by priming sites for subsequent phosphorylation by GSK-3*β*. Therefore, CDK5 is considered to be a therapeutic target for the treatment of AD [16, 17].

Protein kinases have become major screening targets in drug design because these enzymes are involved in all major human diseases. GSK-3*β* and CDK5 are both important in AD pathogenesis. Therefore, these proteins have been extensively used as targets to identify pharmacological inhibitors of potential therapeutic interest. Many CDK5 and GSK-3*β* inhibitors have been identified, most of which act by competing with ATP for binding at the kinase catalytic site. Among these inhibitors, indirubin and its analogs have raised considerable interest. Indirubin isomers have been isolated from marine organisms. The natural product 6-bromoindirubin and its synthetic derivative, 6-bromoindirubin-3′-oxime, display increased selectivity for the inhibition of GSK-3*β* [18, 19]. Moreover, benzazepinones, pyrrolo[2,3-b]pyrazines and 2,6,9-trisubstituted purines all inhibited GSK-3*β* and CDK5 [16, 20].

In this study, we computationally designed multi-target drugs based on the polypharmacology concept, which is currently being actively pursued. Multi-target inhibitors that inhibit with both GSK-3*β* and CDK5 will be beneficial in the prevention and treatment of AD. Previous reports by Li et al. [21] and Olivia et al. [22] provide good perspectives regarding this point. Using a virtual screening method, we screened out novel structures as top leads for AD. 4*H*-benzo[d][1, 3]oxazin-4-one, phthalazin-1(2*H*)-one and 3-hydroxy-1*H*-pyrrol-2(5*H*)-one have dual activity as both GSK-3*β* and CDK5 inhibitors and were designed by computational methods, and these structures are different from those used in previous modeling studies [21]. The drug-like properties of these compounds were predicted. Moreover, we demonstrated that the identified compounds can inhibit A*β*_25-35_-induced neurotoxicity in SH-SY5Y cells.

## RESULTS

### Virtual screening for GSK-3β/CDK5 dual inhibitors

The threshold value of the docking energy should first be determined. Co-crystalized ligands are the best choice for this process. We docked the ligands, phosphoaminophosphonic acid-adenylate ester and (4-amino-2-(4-chlorophenylamino)thiazol-5-yl)(3-nitrophenyl)methanone, in their crystallized conformations to GSK-3*β* and CDK5, respectively, with Autodock 4.2. The docking energies were −10.4 kcal/mol for phosphoaminophosphonic acid-adenylate ester (docked with GSK-3*β*) and −11.1 kcal/mol for (4-amino-2-(4-chlorophenylamino)thiazol-5-yl)(3-nitrophenyl)methanone (docked with CDK5). Therefore, in the virtual screening step, the compounds with docking energies close to −10.4 kcal/mol (GSK-3*β*) and −11.1 kcal/mol (CDK5) could be considered as potential GSK-3*β*/CDK5 dual inhibitors. We finally set −10 kcal/mol (GSK-3*β*) and −11 kcal/mol (CDK5) as the threshold values to retain more diverse structures.

The first search process captured approximately 2,000 compounds that met the screening criteria for GSK-3*β*. These compounds were then docked into CDK5. In this step, 127 compounds were screened out, for which the docking energies for CDK5 were all more negative than −11 kcal/mol. The docking energies of these 127 compounds were provided in Supplementary Table 1.

It is an interesting observation that, for the same compound, the docking energy of GSK-3*β* is more negative than the docking energy of CDK5. This might be caused by structural differences between the ATP-binding sites of GSK-3*β* and CDK5.

The binding modes of the proposed possible dual inhibitors for GSK-3*β* and CDK5 were further analyzed using Autodock 4.2. This program consumes more cpu time, but Autodock 4.2 predicts the binding conformations and the binding energy of each docked compound more accurately than Autodock Vina. Regarding GSK-3*β*/CDK5 docking using Autodock 4.2, most of the compounds scored similar or better binding energy values compared to the results from Autodock Vina. However, some compounds exhibited different binding modes, which might be influenced by the different conformational searching algorithms.

### ClogP and PSA of hits

**Figure 1:**
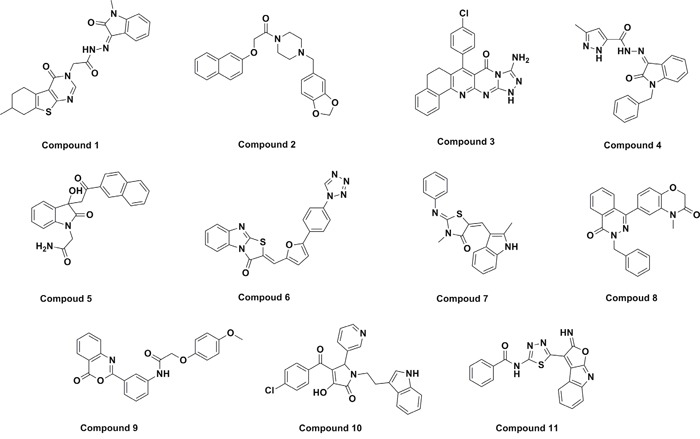
The chemical structures of main hit compounds

Furthermore, we aimed to discover novel scaffolds, which are helpful in the discovery of novel AD drugs. “Drug-likeness” is widely integrated into the early stages of drug discovery. Tools that estimate drug-likeness are valuable in the early stages of lead discovery and can be used to filter out compounds with undesirable properties from screening libraries and to prioritize hits from primary screens. Therefore, it is necessary to predict druglike pharmacokinetic properties. Finally, eleven candidate compounds were carefully selected by considering the docking pose, structural diversity and druglikeness. The eleven hits can be classified into many clusters, including different singletons (Figure 1). The ClogP and PSA of five hits screened out in this study are listed in Table 1.

**Table 1:** Docking energy of five hits

Compounds	ClogP	PAS (Å^2^)	GSK-3*β* Docking Energy (kcal/mol)	CDK5 Docking Energy (kcal/mol)
Compound 05	1.73	100.7	−10.3	−11.1
Compound 06	3.34	116.9	−10.8	−11.5
Compound 08	3.46	62.21	−10.6	−11.6
Compound 09	3.0	86.22	−10.6	−11.0
Compound 10	3.32	86.29	−10.4	−11.1

### Binding modes of the identified hits

**Figure 2:**
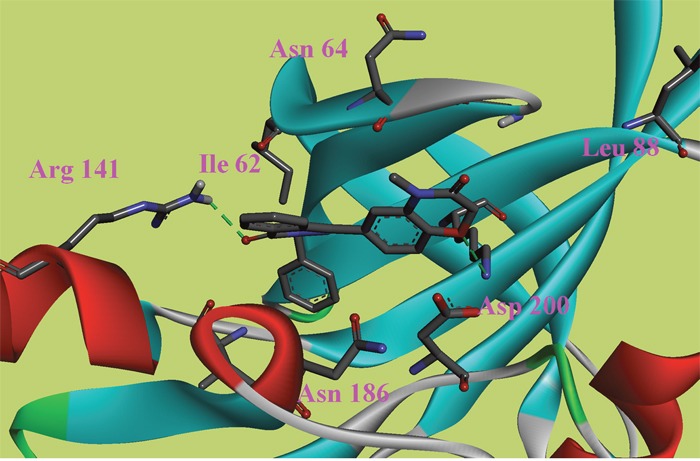
The ATP binding site is represented in ribbon form, and ligands are shown as stick representation, colored by element, H-Bonding interactions are presented with red lines The important interacting residues are shown in stick representation and labeled. Docked Compound 8 with GSK-3*β*.

The binding modes and the molecular interactions of the hits were also compared with that of the inhibitor in the crystal structure of GSK-3*β* (PDB: 1J1B). This compound was considered to be a reference in assessing the binding modes of the hits. The inhibitor in the crystal structure of GSK-3*β* has a more negative binding energy and forms more hydrogen bond interactions, which may be due to the flexible structure of the side chain. The co-crystallized compound in CDK5 (PDB code: 4AU8), (4-amino-2-(4-chlorophenylamino)thiazol-5-yl)(3-nitrophenyl)methanone, also formed hydrogen bond interactions with adjacent residues (Glu81, Lys33, and Cys83). π-π interactions and hydrophobic interactions were also observed. Some distinctive candidates were chosen from among the hits to illustrate the binding results. Most of these compounds have not been reported as inhibitors for any target, and these compounds were chosen on the basis of the observed molecular interactions with the respective important residues in GSK-3*β* and CDK5 (Figure 2–Figure 6). The binding modes and molecular interactions between some of these compounds and the active site components of both the targets are discussed below.

**Figure 3:**
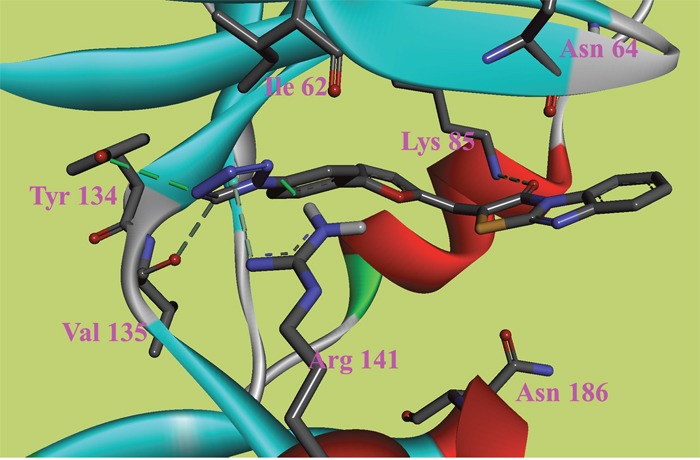
The ATP binding site is represented in ribbon form, and ligands are shown as stick representation, colored by element, H-Bonding interactions are presented with red lines The important interacting residues are shown in stick representation and labeled. Docked Compound 6 with GSK-3*β*.

**Figure 4:**
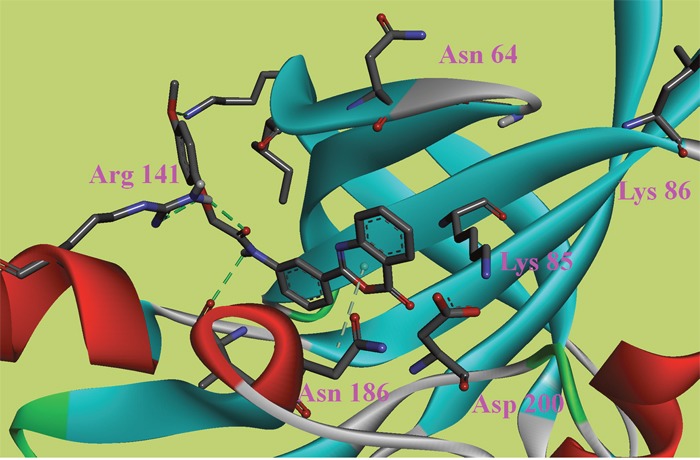
The ATP binding site is represented in ribbon form, and ligands are shown as stick representation, colored by element, H-Bonding interactions are presented with red lines The important interacting residues are shown in stick representation and labeled. Docked Compound 9 with GSK-3*β*.

**Figure 5:**
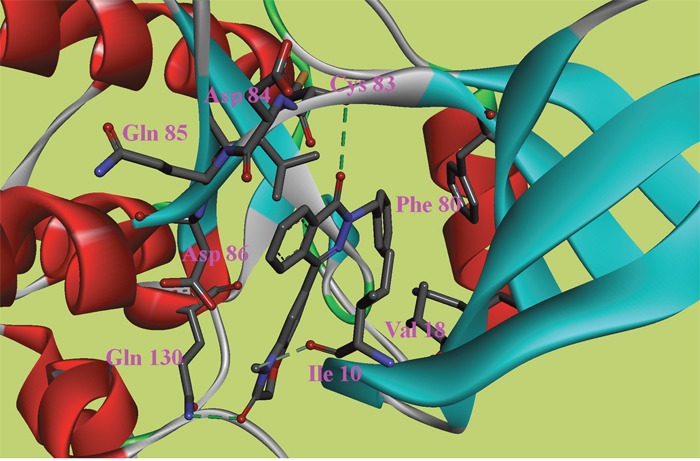
The ATP binding site is represented in ribbon form, and ligands are shown as stick representation, colored by element, H-Bonding interactions are presented with red lines The important interacting residues are shown in stick representation and labeled. Docked Compound 8 with CDK5.

**Figure 6:**
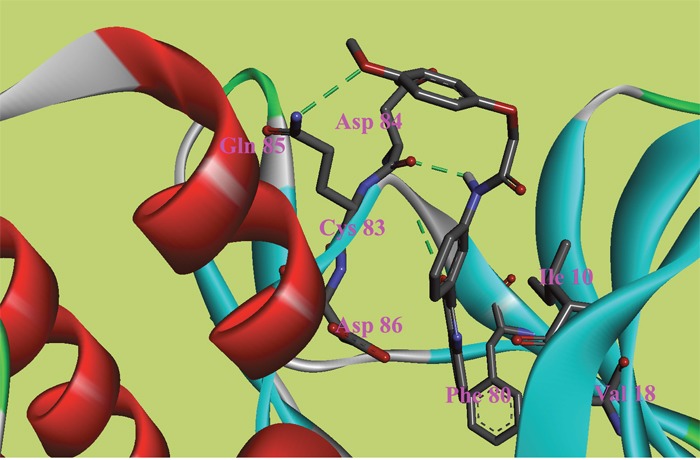
The ATP binding site is represented in ribbon form, and ligands are shown as stick representation, colored by element, H-Bonding interactions are presented with red lines The important interacting residues are shown in stick representation and labeled. Docked Compound 9 with CDK5.

In this study, to gain insight into the potential interactions between the ligands and GSK-3*β*/CDK5, we evaluated all residues within 5 Å of the ligands. In the docking study of GSK-3*β*, compound 6, compound 8 and compound 9 had better binding affinity than the other ligands. The carbonyl group of the diazine ring and the oxygen atom in the morpholine ring acted as hydrogen bond acceptor in compound 8 (Figure 2). Furthermore, Val70 and Ala83 formed π-alkyl interactions and Asp200 formed π-sigma interactions with compound 8. In the 3D docking model, we found that phenyl group in this structure stretched to the hydrophobic pocket in the binding site, which suggests that the hydrophobic pocket formed by Val70, Val110, Leu132, Leu188 and Cys199 is very important for ligand binding affinities. Therefore, the design of new inhibitors targeted to GSK-3*β* should consider the properties of this region. Compound 6 is another ligand with a more negative binding energy for GSK-3*β* (Figure 3). The tetrazole in this structure simultaneously forms two hydrogen bond interactions, a π-positive charge, a π-alkyl and a carbon hydrogen bond interactions. The carbonyl group in the thiazolone ring acted as a hydrogen bond acceptor, and Asp200 had a π-negative charge interaction with the thiazolone ring in this compound. The only amide group in Compound 9 hydrogen bonded with Arg141 and Val135, and hydrogen bond interactions contributed greatly to the binding affinity of this compound (Figure 4). The comparison of these compounds were provided Supplementary Figure 1. Compound 10 hydrogen bonded with Val135, Gln185, Asn186 and Cys199. The hydroxyl group in the structure can form three hydrogen bonds with Gln185, Asn186 and Cys199, respectively. Therefore, this hydroxyl group should be kept in lead optimization process.

In the docking study of CDK5, the verification process for Compound 8 suggested that the carbonyl group of diazine ring was hydrogen-bonded to Cys83, and the predicted H-bond distance was 3.24 Å (Figure 5). The carbonyl group of the morpholine ring in the structure was also hydrogen-bonded to residue Gln130. Val18, Ile10, and Phe80 were also located within 5 Å of Compound 8; however, these three residues were involved in hydrophobic contacts rather than in hydrogen-bonding interactions. As mentioned above, hydrophobic interactions were also identified between Val18, Ile10, Phe80 and Compound 9 (Figure 6); thus, it was obvious that these residues are key residues in CDK5 ligand binding. The oxygen atom of the ester group in Compound 9 acted as a hydrogen bond acceptor for Cys83. The NH of the amide group and the oxygen atom of the methoxyl group interacted with Asp84 and Gln85, respectively. The comparison of these compounds were provided Supplementary Figure 2.

**Figure 7:**
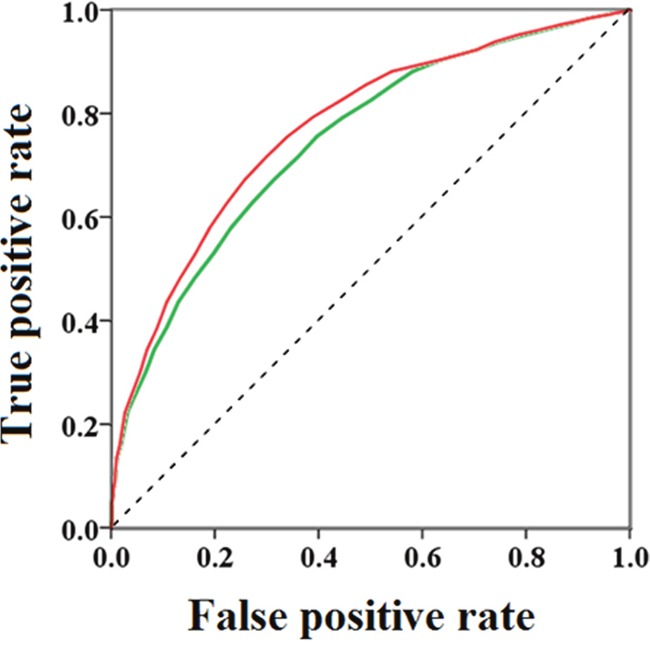
Receiver operating characteristic (ROC) obtained using the property-matched decoy set (Set I) and reported GSK-3*β* inhibitors (Set II): green line for test Set I; red line for test Set I and Set II together

The docking models were found to give considerably good enrichment, suggesting that the models are able to moderately accurately differentiate between inhibitors and decoys. Two sets (Set I and Set II) of compounds were collected together to produce the enrichment data. We did not separately assess Set I because the 200 reported inhibitors formed a small sample size. However, we could not ignore the structural diversity of these compounds, so we combined Set I and Set II. We also chose to analyze the enrichment results obtained from using Set II alone because, as a better match of the molecular properties, compared to Set I, Set II is expected to give unbiased results and provide a good reflection of the actual performance of the screening process. The ROC analysis of Set II had an AUC = 0.747, which is a little smaller than the AUC (0.773) of the two sets (Set I and Set II). A similar trend was observed for the two ROCs. These observations are clearly illustrated by the ROC plots shown in Figure 7.

### The effect of hits on Aβ_25-35_-induced cytotoxicity

In this study, we selected eleven compounds to test their protective effects on the cytotoxicity of A*β*_25-35_ aggregation, Compound 01 – Compound 11. A*β*_25-35_ induces the neurodegeneration of cortical and hippocampal neurons through oxidative stress, secondary excitotoxicity and a wide range of molecular events that disturb neuronal homeostasis. An MTT assay was used to determine the protective effect of the hits on cell death. A*β*_25-35_ was applied to cell cultures at a concentration of 20 μM, and cell survival was assessed 72 h later.

**Figure 8:**
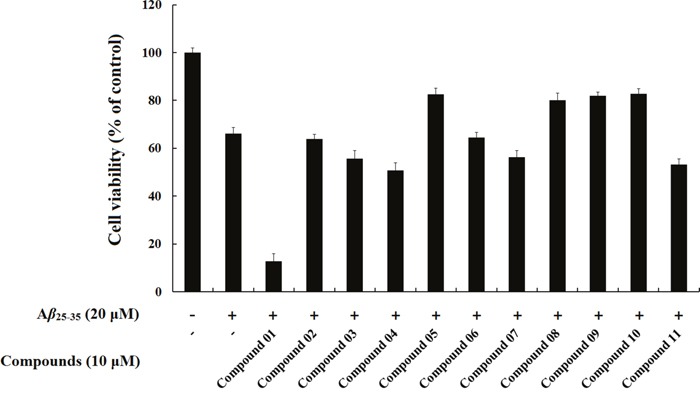
The protective effects of compounds on A*β*_25-35_-induced cell death in SH-SY5Y cells SH-SY5Y cells were exposed to 20 μM A*β*_25-35_ for 72 h in the absence or presence of the compounds. Cells viability was identified using MTT assay. The results were shown as the mean ± SD of three independent experiments. The absorbance of untreated cells was normalized to 100%.

As shown in the Figure 8, 20 μM A*β*_25-35_ significantly decreased (66.16%, *P* < 0.01) the MTT redox potential of the SH-SY5Y cells, whereas Compound 08 had an inhibitory effect on toxicity when the fibrillation of A*β*_25-35_ occurred in its presence, as indicated by the increase in the MTT redox potential in the A*β*_25-35_+ Compound 08 cells (80.07%, *P* < 0.01). The MTT assay showed that Compound 08 treatment reversed the cell viability inhibited by A*β*_25-35_ in SH-SY5Y cells. Compound 09 (82.91%, *P* < 0.01) significantly increased the cell viability compared with A*β*_25-35_. These data revealed that Compound 09 protected SH-SY5Y cells against A*β*_25-35_-induced toxicity. The core structures of Compound 08 and Compound 09 are similar. They both have benzo-heterocycle fragments. Furthermore, the carbonyl group on the six-membered heterocycles of the two compounds are both significant for ligand binding. Compound 05 (82.63%, *P* < 0.05) and Compound 10 (82.80%, *P* < 0.05) can also inhibit the decreased cell viability induced by A*β*_25-35_. Although the chemical structures of Compound 05 and Compound 10 are mainly different, they share the same core structure, pyrrolidin-2-one. This structure is a common fragment in kinase inhibitors and a known GSK-3*β* inhibitor BIP-135 has this fragment. Pyrrolidin-2-one is a rigid five-member ring and the carbanyl group in this structure usually forms hydrogen bond. Of the eleven tested compounds, compound 01 decreased cell viability (12.80%, *P* < 0.05). We infer that compound 01 synergy with A*β*_25-35_ to induce cytotoxicity.

The objective of the vitro experiment was to evaluate whether screened compounds can successfully inhibit A*β*_25-35_-induced toxicity in the human neuroblastoma cells. There is currently no direct experimental evidence to support the effect of 4*H*-benzo[d][1, 3]oxazin-4-one derivatives, phthalazin-1(2*H*)-one derivatives and 3-hydroxy-1*H*-pyrrol-2(5*H*)-one derivatives on neurotoxicity and morphological deterioration. Thus, to test our hypothesis, we directly conducted A*β*_25-35_ fibrillation in cell culture media in the presence of Compound 08 and Compound 09 to determine whether these compounds have a positive effect on neurotoxicity. Furthermore, cell viability did not decrease after exposure to these compounds, which suggests a good safety profile.

## DISCUSSION

In summary, a molecular docking screening strategy was applied to meet the critical challenges faced in designing efficient multi-target drugs to treat AD. Initially, a database of small molecules was docked with GSK-3*β* using the widely accepted molecular docking program Autodock Vina, and the compounds that had good binding characteristics were selected for docking to the second target, CDK5. Key residues in GSK-3*β* and CDK5 were identified. To confirm the binding modes of the compounds, we calculated the binding energies of selected possible dual hits using Autodock 4.2.

We considered the accuracy of the docking study by calculating the area under the ROC curve. The result suggested that our docking model can clearly identify inhibitors within a database. Moreover, the ability of a compound to penetrate the BBB is significant for drugs that treat AD. Therefore, we also calculated the ClogP and PSA of the candidate compounds and found that most of these compounds have good BBB penetrating ability.

The compounds with better binding characteristics were selected as possible dual inhibitors of GSK-3*β* and CDK5 to use in blocking tau protein phosphorylation. Reverse validation also suggested the same compounds as possible dual inhibitors. In conclusion, five hits were screened as multi-targeted agents for the treatment of AD. Among these compounds, compound 05, compound 08, compound 09 and compound 10 gave the best results and are potential agents for the treatment of AD. These findings will be instrumental for rational design of drug candidates for AD.

## MATERIALS AND METHODS

### Virtual screening

We used an efficient molecular docking software, Autodock Vina [23], to select more diverse active compounds. We screened 181,057 compounds from the Specs library with our docking model for GSK-3*β*/CDK5 inhibitors as the query. In the virtual screening step, the docking score is the binding energy and the best docking mode is considered the conformation with the more negative binding energy [24, 25].

The crystal structures of GSK-3*β* (PDB code: 1J1B) and CDK5 (PDB code: 4AU8) were retrieved from the Protein Data Bank [26, 27]. In the PDB file, GSK-3*β* was co-crystallized with a small molecule, phosphoaminophosphonic acid-adenylate ester, interacting with Asp133, Val135, Arg141, Gln185, and Lys85. The CDK5 PDB file contains the coordinates for the monomers B (CDK5) and E (p25). The compound (4-amino-2-(4-chlorophenylamino)thiazol-5-yl)(3-nitrophenyl)methanone was co-crystallized with the two proteins, and this compound interacts with Glu81, Lys33, and Cys83. The selected structure of CDK5 contains missing residues in the vicinity of the active site, and we constructed all the missing residues in the CDK5 structure using Build Homology Models in Discovery Studio 2.5. The PDB files were processed with Discovery Studio 2.5. All the hydrogen atoms were added, followed by ensuring that multiple bond orders were correctly defined and that the hydrogen atoms were properly added for all amino acids. The co-crystallized ligand, the solvent water molecules and other cofactors were removed.

The model of the protein was converted to a PDBQT format using AutoDock Tools 1.5.6; Kollman united atom partial charges were then assigned for the receptor. The grid size of the search space was set at 40 Å × 40 Å × 40 Å centered on the binding site, with a default grid-point spacing of 0.375 Å.

Our strategy to discover dual-target inhibitors began with the preparation of the ligand database, which was then docked with the active site of a target protein, thus predicting the binding conformations and molecular interactions. Based on a binding-mode analysis, compounds with suitable binding characteristics that exhibited strong interactions with key residues at the active site of the first target (GSK-3*β*) were chosen for further screening. In the next step, selected compounds were docked with the active site of the second target (CDK5) to examine the binding affinities of these compounds. The binding modes of all the docked compounds were analyzed according to their molecular interactions. The compounds that displayed strong key interactions at both of the binding sites were considered potential GSK-3*β*/CDK5 dual hits. The compounds with high scores were docked again to both target proteins using a different molecular docking program (AutoDock 4.2) to verify the accuracy of the screening results [24]. For the Autodock 4.2 verification process, the Lamarckian genetic algorithm was used with a population size of 200 dockings and 25 million energy evaluations. The results were clustered according to the root-mean-square deviation (RMSD) criterion. The final result was determined based on a combination of clustering, energy and interacting residue data.

Enrichment studies were carried out to test the ability of the GSK-3*β* docking model to differentiate inhibitors from decoys. Therefore, additional docking studies were performed on 200 reported GSK-3*β* inhibitors (Set I), and a set of structures (Set II) was selected based on molecular properties from the ZINC database [28–35]. There were 877 ligands in Set II, which was non-randomly selected from the ZINC database [36]. We followed the rules below to select the molecules of Set II: a molecular weight from 350 to 500, an XLogP value from 1 to 4, a hydrogen-bond-donor count from 1 to 5, a hydrogen-bond-acceptor count from 5 to 10 and a TPSA from 0 to 90. These parameters would indicate favorable drug-like properties. Compared with previously reported inhibitors, Set II had more closely matched chemical properties and was considered to contain efficient challengers for the inhibitors. The inhibitors retrieved from the literature were prepared using Discovery Studio 2.5 to assign appropriate protonation states [37], generate tautomers and optimize the geometry prior to the docking calculations. Receiver operating characteristic (ROC) curves were prepared by plotting the true-positive rate against the false-positive rate. The ROC area under the curve (AUC) value was calculated to measure the early recognition performance and the overall predictive performance.

### Physicochemical properties and predicted blood-brain barrier (BBB) permeability of CDK5-GSK-3β inhibitors

The BBB is a highly selective permeability barrier that separates the circulating blood from the brain extracellular fluid in the CNS. The BBB is the bottleneck in AD drug development and is the most important factor limiting the future growth of neurotherapeutics. Therefore, the likelihood that candidate compounds can cross the BBB should be predicted.

The CLogP (octanol/water partition coefficient) and polar surface area (PSA) are crucial indicators of BBB permeability [38, 39]. The CLogP of the hits were calculated using the program Bio-Loom for Windows, Version 5. The PSA was determined using the web tool mprop (http://www.molsoft.com/mprop/).

### Cell culture and drug treatment

A*β*_25–35_ (Aladdin, Shanghai, China) was dissolved in water to obtain a 1 mM stock solution. Aliquots were stored at −20°C and thawed at 37°C for 5-7 days for use. The human neuroblastoma cell line SH-SY5Y was kindly donated by Department of Pharmacology of Harbin Medical University. Cells were cultured in RPMI-1640 supplemented with 10% FBS at 37°C. The cells were grown at 37°C in a humid 5% CO_2_ environment, and the medium was routinely replaced every 2 days. Cells were treated with 20 μM A*β*_25–35_ or 20 μM A*β*_25–35_ with 10 μM compound. Control cells were cultured under normal conditions.

### Cell viability assay

Cells were plated in 96-well plates containing complete medium and cultured for 24 h. The cells were then treated with compounds at the indicated concentrations for specified times. After drug treatment, cell viability was measured using the MTT assay. Briefly, 10 μL of MTT solution (5 mg/mL) was added to each well and incubated for 4 h at 37°C. After removing the supernatant, 100 μL DMSO was added into each well. The absorbance was measured at 490 nm. All experiments were repeated 3 times; 10 μM compound (dissolved in DMSO) was added to the cultures 1 h prior to the 24h A*β*_25-35_ exposure.

## SUPPLEMENTARY MATERIALS FIGURES AND TABLES




