# Supplementary material for: Designing of dual inhibitors for GSK-3β and CDK5: Virtual screening and in vitro biological activities study
Source: Oncotarget. 2017 Feb 4;8(11):18118–28. doi: 10.18632/oncotarget.15085 (PMC5392312; doi:10.18632/oncotarget.15085)
Supplement: Supplementary file 1 [file oncotarget-08-18118-s001.pdf]

Designing of dual inhibitors for GSK-3*β* and CDK5: Virtual screening and *in vitro* biological activities study

SUPPLEMENTARY FIGURES AND TABLE

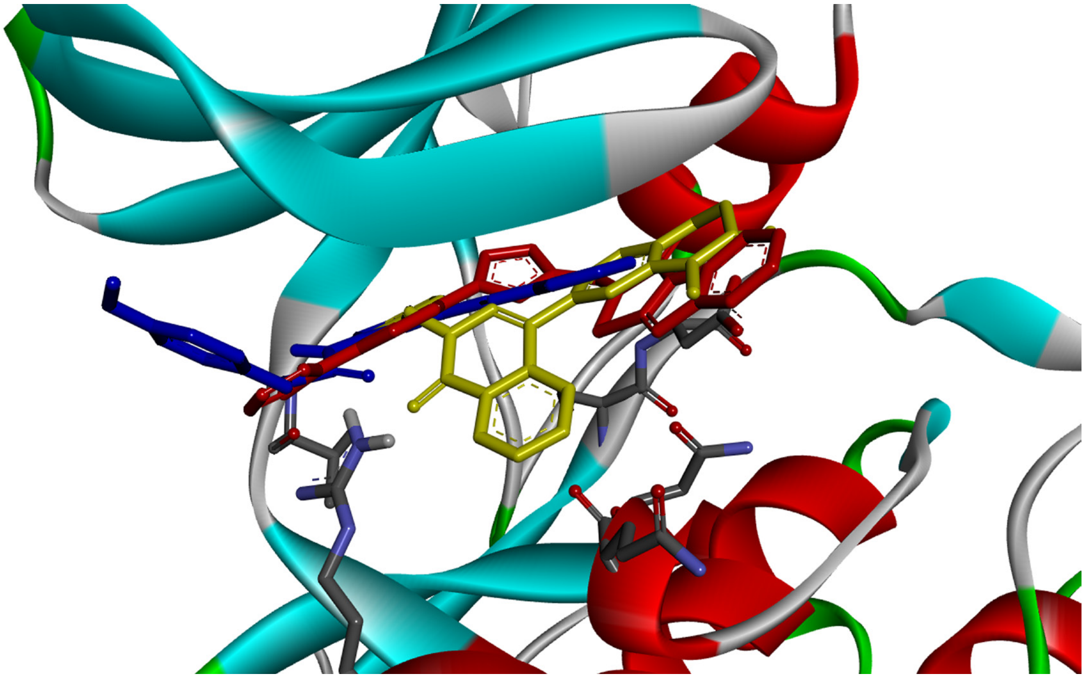

Supplementary Figure 1: The binding conformations of Compound 6, Compound 8 and Compound 9 for GSK-3β.

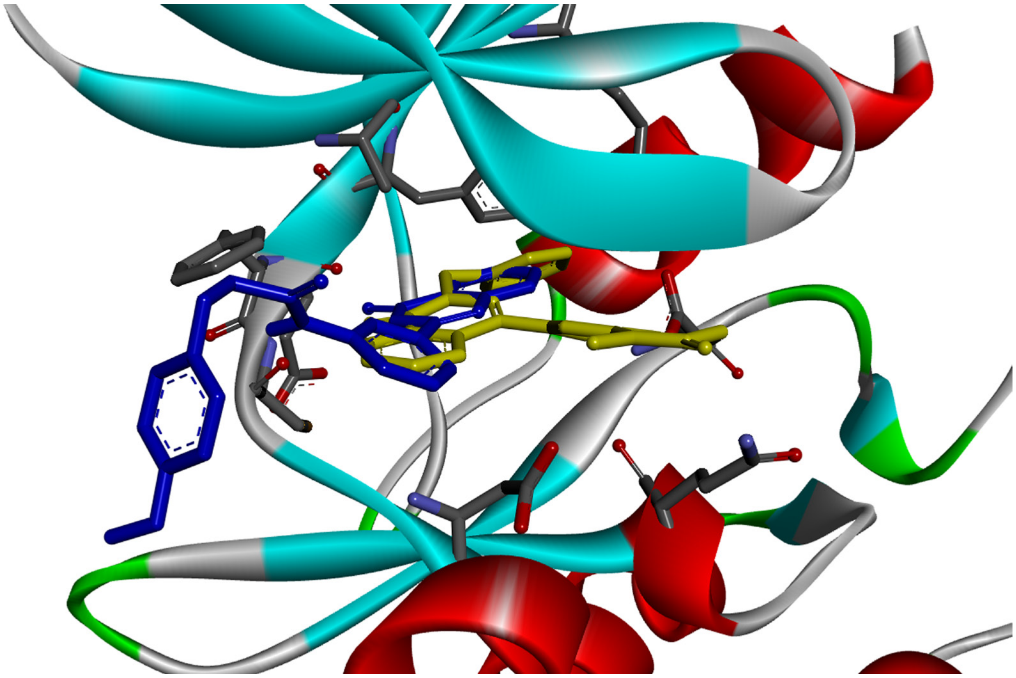

Supplementary Figure 2: The binding conformations of Compound 8 and Compound 9 for CDK5.

Supplementary Table 1: Docking energies of 127 compounds

See Supplementary File 1
