# Supplementary material for: Designing of dual inhibitors for GSK-3β and CDK5: Virtual screening and in vitro biological activities study
Source: Oncotarget. 2017 Feb 4;8(11):18118–28. doi: 10.18632/oncotarget.15085 (PMC5392312; doi:10.18632/oncotarget.15085)
Supplement: Supplementary file 2 [file oncotarget-08-18118-s002.doc]

**Supplementary Table1 Docking energies of 127 compounds**

| Compounds | GSK-3*β* Docking Energy (kcal/mol) | CDK5 Docking Energy (kcal/mol) |
| --- | --- | --- |
| Compound 01 | -10.5 | -11.5 |
| Compound 02 | -10.7 | -11.2 |
| Compound 03 | -10.8 | -11.7 |
| Compound 04 | -10.5 | -11.6 |
| Compound 05 | -10.3 | -11.1 |
| Compound 06 | -10.8 | -11.5 |
| Compound 07 | -10.9 | -11.2 |
| Compound 08 | -10.6 | -11.6 |
| Compound 09 | -10.6 | -11 |
| Compound 10 | -10.4 | -11.1 |
| Compound 11 | -10.7 | -11.3 |
| s_1_1016 | -11 | -11.4 |
| s_1_1011 | -10.8 | -11 |
| s_1_1326 | -11.3 | -11.2 |
| s_1_1691 | -11.2 | -11.4 |
| s_1_290 | -10.8 | -11.2 |
| s_1_944 | -10.4 | -11.7 |
| s_1_947 | -10.7 | -11.6 |
| s_1_961 | -10.6 | -11.2 |
| s_3001_1213 | -10.4 | -11.4 |
| s_3001_1189 | -10.8 | -11.1 |
| s_3001_1275 | -10.8 | -11 |
| s_6001_212 | -11.3 | -11.4 |
| s_6001_240 | -10.4 | -11.2 |
| s_6001_242 | -11.6 | -11.2 |
| s_6001_711 | -10.9 | -11.7 |
| s_9001_2 | -10.4 | -11.8 |
| s_9001_4 | -10.7 | -12 |
| s_9001_631 | -10.8 | -11.3 |
| s_12001_130 | -10.7 | -11.7 |
| s_12001_409 | -10.8 | -11.1 |
| s_15001_97 | -11 | -12.1 |
| s_15001_127 | -10.9 | -11.1 |
| s_18001_56 | -10.8 | -11.6 |
| s_18001_120 | -10.6 | -11.4 |
| s_18001_386 | -10.8 | -11.3 |
| s_21001_209 | -10.7 | -11.4 |
| s_21001_399 | -10.6 | -11 |
| s_27001_431 | -11.7 | -11.4 |
| s_27001_610 | -10.6 | -11.1 |
| s_30001_160 | -10.7 | -11.4 |
| s_30001_331 | -10.5 | -12.1 |
| s_33001_553 | -11.4 | -11.4 |
| s_33001_750 | -10.5 | -11.1 |
| s_39001_804 | -10.9 | -11.6 |
| s_39001_893 | -10.6 | -11.8 |
| s_42001_1001 | -10.8 | -11.5 |
| s_42001_1027 | -10.8 | -11.3 |
| s_42001_1037 | -10.7 | -11 |
| s_42001_1040 | -10.6 | -11.2 |
| s_42001_634 | -10.7 | -11.2 |
| s_42001_639 | -11.7 | -11.1 |
| s_42001_717 | -10.6 | -11 |
| s_42001_80 | -11.7 | -11.1 |
| s_45001_11 | -10.6 | -11.5 |
| s_45001_106 | -10.5 | -11 |
| s_51001_111 | -11 | -11.6 |
| s_51001_1198 | -10.6 | -11 |
| s_57001_101 | -10.8 | -11.5 |
| s_57001_102 | -10.5 | -11.4 |
| s_57001_1258 | -11.3 | -11 |
| s_57001_670 | -10.9 | -11.2 |
| s_60001_330 | -10.5 | -11.5 |
| s_60001_461 | -10.6 | -11.7 |
| s_60001_774 | -10.5 | -11.3 |
| s_60001_778 | -10.7 | -11 |
| s_60001_856 | -10.5 | -11 |
| s_63001_103 | -10.6 | -11.5 |
| s_63001_212 | -10.4 | -11.1 |
| s_69001_251 | -10.9 | -11.6 |
| s_69001_448 | -10.4 | -11 |
| s_72001_1023 | -11.2 | -11.5 |
| s_72001_667 | -11.1 | -11.3 |
| s_75001_31 | -10.8 | -11.5 |
| s_75001_655 | -10.4 | -11.6 |
| s_75001_657 | -10.5 | -11.5 |
| s_75001_726 | -11 | -11.2 |
| s_78001_21 | -10.4 | -11.5 |
| s_78001_26 | -10.6 | -11 |
| s_78001_730 | -10.4 | -11.4 |
| s_90001_1401 | -10.8 | -11.1 |
| s_90001_165 | -10.6 | -11 |
| s_93001_210 | -10.4 | -11.1 |
| s_93001_380 | -11.9 | -11.2 |
| s_93001_869 | -10.4 | -11 |
| s_105001_491 | -10.6 | -11.1 |
| s_105001_782 | -10.8 | -11.3 |
| s_108001_1042 | -10.4 | -11.4 |
| s_108001_1043 | -10.8 | -11 |
| s_108001_939 | -10.4 | -11.2 |
| s_114001_138 | -10.8 | -11.6 |
| s_114001_1447 | -10.3 | -11.3 |
| s_117001_107 | -11.5 | -11 |
| s_117001_519 | -10.8 | -11.2 |
| s_120001_846 | -10.8 | -11.7 |
| s_120001_926 | -11 | -11.9 |
| s_120001_945 | -10.8 | -11.2 |
| s_123001_487 | -10.6 | -11.3 |
| s_123001_631 | -10.7 | -11.4 |
| s_126001_248 | -10.3 | -11.7 |
| s_126001_341 | -10.9 | -11 |
| s_126001_375 | -10.8 | -11.1 |
| s_138001_876 | -10.3 | -11.7 |
| s_138001_95 | -10.6 | -11.3 |
| s_138001_99 | -10.3 | -11.2 |
| s_141001_129 | -10.7 | -11.7 |
| s_141001_1360 | -10.7 | -11 |
| s_144001_105 | -10.9 | -11.3 |
| s_144001_1442 | -10.7 | -11.1 |
| s_144001_162 | -10.3 | -11.1 |
| s_144001_605 | -10.6 | -11.3 |
| s_144001_885 | -10.3 | -11.1 |
| s_147001_13 | -10.8 | -11.3 |
| s_147001_1735 | -10.7 | -11 |
| s_156001_151 | -10.6 | -11.7 |
| s_156001_1066 | -10.6 | -11.2 |
| s_156001_1068 | -10.6 | -11.1 |
| s_156001_1075 | -10.9 | -11 |
| s_156001_1076 | -10.8 | -11 |
| s_156001_1079 | -11.1 | -11 |
| s_156001_1094 | -11.1 | -11.3 |
| s_156001_1097 | -11.1 | -11.2 |
| s_156001_1098 | -11.3 | -11.5 |
| s_156001_1099 | -10.8 | -11.5 |
| s_156001_1153 | -10.7 | -11.5 |
| s_156001_1156 | -10.6 | -11.7 |
| s_159001_1160 | -10.4 | -11.3 |
